# Iodine status in Norwegian preschool children and associations with dietary iodine sources: the FINS-KIDS study

**DOI:** 10.1007/s00394-018-1768-0

**Published:** 2018-07-04

**Authors:** Ive Nerhus, Mathilde Odland, Marian Kjellevold, Lisa Kolden Midtbø, Maria Wik Markhus, Ingvild Eide Graff, Øyvind Lie, Ingrid Kvestad, Livar Frøyland, Lisbeth Dahl, Jannike Øyen

**Affiliations:** 1 Institute of Marine Research (IMR), Nordnes, P.O. Box 1870, 5817 Bergen, Norway; 2 Clinical Institute 1, University of Bergen, Bergen, Norway; 3 Regional Centre for Child and Youth Mental Health and Child Welfare, Uni Research Health, Bergen, Norway; 4 Present Address: Uni Research Health, Bergen, Norway; 5 Present Address: Directorate of Fisheries, Bergen, Norway

**Keywords:** Creatinine, Fish, Iodine, Milk, Preschoolers, Urinary iodine concentration

## Abstract

**Purpose:**

Iodine is an essential trace element necessary for thyroid hormone synthesis. Iodine deficiency is a continuing public health problem despite international efforts to eliminate it. Studies on iodine status in preschoolers are scarce. Thus, the aims of the current study were to determine the iodine status and to investigate possible associations between urinary iodine concentration (UIC) and estimated 24 h iodine extraction (UIE) and iodine-rich foods.

**Methods:**

Data are cross-sectional baseline data, obtained from the two-armed randomized controlled dietary trial “Fish Intervention Studies-KIDS” (FINS-KIDS) conducted in Bergen, Norway. UIC was determined by inductively coupled plasma-mass spectrometry in spot urine samples. Inadequate UIC was defined as median < 100 µg/L, and low estimated 24 h UIE as < 65 µg/day. Habitual dietary intake was assessed by a short food frequency questionnaire. Logistic regression models were used to investigate possible associations between UIC and estimated 24 h UIE and iodine-rich dietary sources including seafood, dairy products and eggs. Iodine/creatinine ratio (I/Cr) was also estimated.

**Results:**

Urinary spot samples were obtained from 220 children. The median (interquartile range) UIC and estimated 24 h UIE was 132 (96) µg/L, and 65 (55) µg/day, respectively. The majority of children had an estimated I/Cr ratio within 100–199 µg/g. Intake of sweet milk < 2 times/day versus ≥ 2 times/day was associated with UIC < 100 µg/L (OR 2.17, 95% CI 1.07–4.38, *p* = 0.031). Intake of dairy products (OR 3.59, 95% CI 1.13–11.43, *p* = 0.031) and sweet milk (OR 2.77, 95% CI 1.37–5.61, *p* = 0.005) < 2 times/day versus ≥ 2 times day was associated with estimated 24 h UIE < 65 µg/day.

**Conclusions:**

The preschoolers had adequate iodine status. Low intake of sweet milk and dairy products were associated with low iodine status.

## Introduction

Iodine is a trace element located mainly in the thyroid gland and is essential for thyroid hormone production [1, 2]. Worldwide, iodine deficiency is a continuing public health concern even though salt iodization programs have had a large impact on global iodine nutrition [3]. The iodine content of most foods is low. The only good natural source of iodine is seawater fish and other marine products. However, the main sources of iodine in Norway are milk and dairy products [4, 5]. Europe is still the continent with the highest prevalence of iodine deficiency and 44% of schoolchildren have inadequate iodine status, defined as median urinary iodine concentration (UIC) < 100 µg/L according to the World Health Organization (WHO) [6]. When iodine intakes are inadequate, the body may respond with impaired thyroid hormone synthesis, which can result in several functional and developmental abnormalities [7]. In preschool children, mild-to-moderate iodine deficiency may induce adverse health effects, including impaired intellectual function and physical growth [8, 9].

A high consumption of milk and dairy products have led to eradication of endemic goiter in Norway and Britain since the start of iodine fortification of cow feed in the 1950s [4, 10]. Until recently, Norway has been considered to have an overall adequate iodine status [3]. In a previous study on the iodine content of Norwegian foods along with assessment of iodine intakes in subgroups of the Norwegian population, the iodine intakes among 4-year-old girls and boys were calculated to 98 and 101 µg/day, respectively [5]. This indicates iodine intakes slightly above the recommended intake level for this age group (2–5 years; 90 µg/day) [5]. Recent studies indicate that subgroups within the Norwegian population, including pregnant women and adolescent girls, may suffer from mild iodine deficiency due to inadequate intakes of dietary iodine [4, 5, 11, 12].

UIC in spot urine samples is recommended by WHO, UNICEF (United Nations Children’s Fund) and IGN (Iodine Global Network) for assessing a population’s iodine status. To obtain a more accurate indicator of iodine levels than spot urinary iodine concentration, the urine iodine-to-creatinine ratio (I/Cr) is often used. Creatinine is a breakdown product of creatine and is steadily excreted through urine in 24 h if fluid intake is regular [13]. Excretion of creatinine is constant at ~ 1 g/day, but can vary according to factors such as gender, age and nutritional status [13, 14]. Thus, the iodine concentration measured in spot urine samples can be expressed as µg iodine/g creatinine.

The main aims of this study were to evaluate the iodine status by UIC, I/Cr and estimated 24 h urinary iodine excretion (UIE), and to investigate possible associations between UIC and estimated 24 h UIE and iodine-rich food sources in Norwegian preschool children aged 4–6 years.

## Materials and methods

### Ethics statement

The procedures were in accordance with the Declaration of Helsinki. The children’s caregivers signed a written, informed consent prior to project start. The trial was approved by the Regional Committees for Medical and Health Research Ethics North (2014/1396), and registered in ClinicalTrials.gov (NCT02331667).

### Study procedure and participants

Data in this study are obtained from the two-armed randomized controlled trial, Fish Intervention Studies-KIDS (FINS-KIDS), conducted in Bergen, Norway, from January to June 2015. The overall design, including study enrollment, randomization and other results have been published elsewhere [15]. The children were recruited from kindergartens in the area of Bergen.

### Outcome measures

#### Iodine status

Spot urine samples were collected mainly in each child’s home and delivered to the respective kindergartens. For some children, the samples were collected in the kindergartens and thereafter retrieved by study personnel. Time of sampling or whether they were fasting was not registered. From the kindergartens, the samples were transported to IMR and stored, first at – 80 °C, thereafter at – 20 °C, prior to analysis of UIC by inductively coupled plasma mass spectrometry (ICP-MS). Method description of ICP-MS is previously described by Dahl et al. [16]. In addition to UIC, I/Cr ratio and estimated 24 h UIE was determined. Determination of urinary creatinine concentration was analyzed using the MAXMAT PL II multidisciplinary diagnostic platform with creatinine PAP kit [17]. The I/Cr ratio was determined by dividing the iodine concentration (µg/L) on the creatinine concentration (g/L). Estimation of 24 h UIE was determined using an anthropometry-based reference value for 24 h urinary creatinine excretion (g/day) originally developed in German children [18]. The reference value was determined according to definite height groups. Values from the German study were linked to the median height of the present study population (115 cm).

#### Dietary intake

A revised version of a food frequency questionnaire (FFQ) [19–21] also including information regarding demographics, was distributed electronically to the caregivers. The FFQ contained questions on habitual consumption of fish and seafood items either for dinner or as bread spread, in salads or as a snack meal. In addition, it contained one question regarding habitual intake of dairy products, and one question regarding habitual intake of eggs. Dairy products were summed up in the categories “milk”, “cultured milk” and “yogurt”, “white cheeses” and “brown cheese”. The parents also reported the use of supplement in addition to type and dosage. The children’s use of multimineral supplement were checked for possible iodine content.

### Statistical analyses

The current practice is to report iodine as median concentration (µg/L), due to generally skewed distributed data [22]. Categorical variables are summarized as numbers (percentage) and continuous variables as median with interquartile range (IQR). Independent samples *T* test (age, weight, height, and parental education) and Person Chi-square test (family income) were used for comparisons between boys and girls for demographic variables and independent samples Mann–Whitney *U* test was used for the dietary variables, UIC, creatinine concentration, iodine/creatinine ratio and estimated 24 h UIE.

Unadjusted and adjusted (gender and parental education) logistic regression analyses were used to investigate possible associations of UIC and estimated 24 h UIE with iodine-rich dietary sources (seafood (dinner and spread), dairy products (milk, cultured milk, cheese and egg). Interactions between UIC and gender were added in a final model. Inadequate UIC was defined as median < 100 µg/L [6], and low estimated 24 h UIE < 65 µg/day, corresponding to median estimated 24 h UIE in the included children. The cut-off values for “low” and “high” intake (times per week) of the different food items are given in Table 3.

Two-tailed *p* values < 0.05 were considered statistically significant. Statistical analyses were performed using Statistical Package for the Social Sciences (SPSS^®^ Statistics Version 24).

## Results

### Study population

A total of 314 eligible children where invited, and 232 agreed to participate. Of these, baseline levels of UIC and creatinine were available in 220 of the participants, and 198 of the caregivers answered a food frequency questionnaire (FFQ) on the children’s eating habits at baseline.

Characteristics of participants are presented in Table 1. The median (IQR) age, weight and height of the children was respectively 5.2 (0.9) years, 20.0 (4.0) kg and 115.0 (2.5) cm. The boy’s caregivers had more years of education and higher family income. Five children (2.4%) took daily doses of iodine-containing supplements.

**Table 1:** Characteristics of the participants

	*N*	All (*N* = 220)	Boys (*N* = 106)	Girls (*N* = 114)	*p* value
Demographics					
Age (years)	220	5.2 (0.9)	5.2 (1.1)	5.2 (0.8)	0.161
Weight (kg)	176	20.0 (4.0)	20.0 (5.0)	20.0 (4.0)	0.177
Height (cm)	179	115.0 (8.0)	116.0 (12.0)	113.0 (7.0)	0.487
Education parents (years)	193	16.0 (2.5)	16.5 (2.0)	15.0 (2.0)	0.023
Family income (NOK^a^), *N* (%)	202				
< 200,000 to 549,999		33 (16.3)	6 (6.4)	27 (25.0)	0.001
550,000 to 999,999		81 (40.1)	39 (41.5)	42 (38.9)	
1,000,000 to > 2,000,000		88 (43.6)	49 (52.1)	39 (36.1)	

Data are given as median (IQR) if not other is indicated

*p* value for comparison between boys and girls is given with independent samples *T* test (demographics), Person Chi-square test (family income)

*IQR* interquartile range

^a^100 NOK = approximately 10€/11$

### Urinary iodine status

**Fig. 1:**
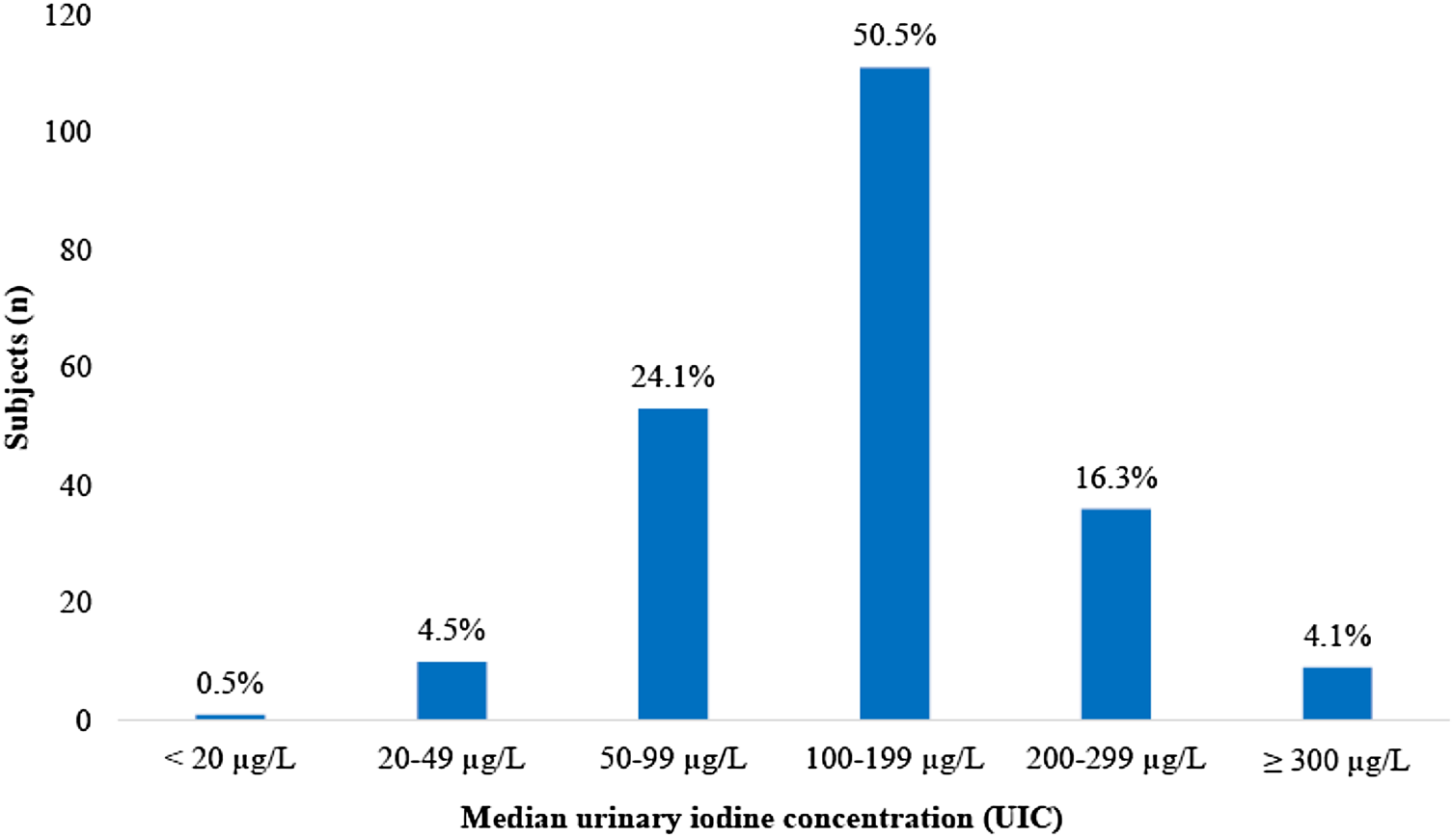
Distribution of urinary iodine concentrations among the children (*N* = 220). Presented according to World Health Organization (WHO)/United Nations Children’s Fund (UNICEF)/International Council for Control of Iodine Deficiency Disorders (ICCIDD) criteria on iodine nutrition in populations (based on surveys in school-aged children ≥ 6 years old)

**Fig. 2:**
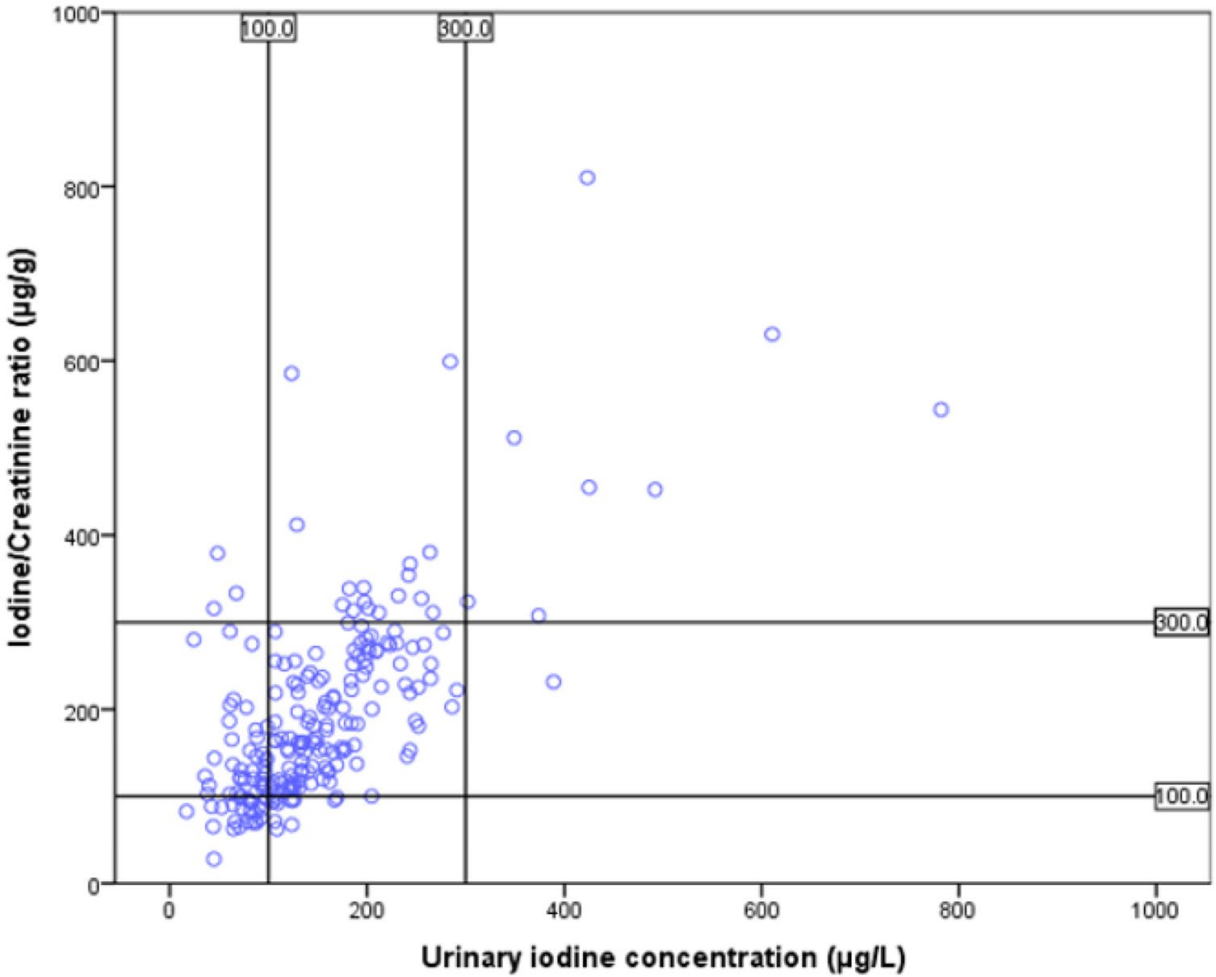
Distribution of urinary iodine concentration and estimated iodine/creatinine ratio among the children and how they are classified in relation to one another (*N* = 220). Each dot represents one child

UIC and creatinine concentration along with the estimated I/Cr ratio and estimated 24 h UIE are given in Table 2. The median (IQR) UIC was 132 µg/L (96), ranging from 17 to 782 µg/L, and median estimated 24 h UIE was 65 (55) µg/day, ranging from 11 to 324 µg/day. There were no significant differences between boys and girls in any of the presented parameters (Table 2). The UIC results presented according to the cut offs for describing population iodine status given by WHO, are shown in Fig. 1. Further, Fig. 1 illustrates that 50.5% of children had UIC within the optimal range (median 100–199 µg/L). In addition, 37 (16.8%) of the children had had an estimated I/Cr ratio below 100 µg/g and 96 (43.6%) within 100–199 µg/g (Fig. 2).

**Table 2:** Urinary iodine and creatinine concentration in the participants

	All (*N* = 220)	Boys (*N* = 106)	Girls (*N* = 114)	*p* value
UIC (µg/L)	132 (96)	136 (97)	131 (97)	0.321
Creatinine concentration (g/L)	0.8 (0.4)	0.8 (0.5)	0.8 (0.4)	0.119
Iodine/creatinine ratio (µg/g)	163 (138)	163 (143)	163 (138)	0.676
Estimated 24 h UIE (µg/day)	65 (55)	65 (57)	65 (55)	0.676

Data are given as median (IQR)

*p* value for comparison between boys and girls is given with independent samples Mann–Whitney *U* test

*IQR* interquartile range, *SD* standard deviation, *UIC* urinary iodine concentration, *UIE* urinary iodine excretion

### Urinary iodine in relation to dietary intake

The odds ratio (OR) for UIC < 100 µg/L, according to the intake of seafood, dairy products and eggs are shown in Table 3. Intake of sweet milk < 2 versus ≥ 2 times/day was associated with UIC < 100 µg/L (OR 2.17, 95% CI 1.07–4.38, *p* = 0.031). This association was similar after adjusting for gender and parental education as shown in Table 3. No other associations between UIC and iodine-rich dietary sources were observed. Interactions between UIC and gender were added in a final model with no significant findings (data not shown).

**Table 3:** Odds ratio (OR) for urinary iodine concentration (UIC) < 100 µg/L according to intake of seafood, dairy products and eggs

Dietary products	*N*	Unadjusted		Adjusted for gender, parental education	
		OR (95% CI)	*p* value	OR (95% CI)	*p* value
Seafood dinner (all)					
< 2 times/week	103	1.22 (0.65–2.28)	0.542	1.27 (0.67–2.43)	0.468
≥ 2 times/week	95	1 (ref.)		1 (ref.)	
Seafood (spreads)					
< 2 times/week	132	1.26 (0.64–2.49)	0.499	1.29 (0.64–2.58)	0.479
≥ 2 time/week	66	1 (ref.)		1 (ref.)	
Dairy products (all)					
< 2 times/day	102	1.96 (0.72–5.54)	0.185	2.02 (0.71–5.78)	0.189
≥ 2 times/day	96	1 (ref.)		1 (ref.)	
Sweet milk					
< 2 times/day	45	2.17 (1.07–4.38)	0.031	2.21 (1.08–4.49)	0.029
≥ 2 times/day	153	1 (ref.)		1 (ref.)	
Cultured milk and yoghurt					
< 4 times/week	90	1.16 (0.62–2.17)	0.641	1.22 (0.64–2.35)	0.542
≥ 4 time/week	108	1 (ref.)		1 (ref.)	
White cheese					
< 4 times/week	94	0.95 (0.51–1.78)	0.871	1.04 (0.55–1.98)	0.901
≥ 4 time/week	105	1 (ref.)		1 (ref.)	
Brown cheese					
< 4 times/week	153	1.21 (0.56–2.60)	0.628	1.28 (0.58–2.83)	0.545
≥ 4 time/week	45	1 (ref.)		1 (ref.)	
Eggs					
< 2 times/week	117	1.22 (0.59–2.13)	0.723	1.28 (0.66–2.49)	0.463
≥ 2 times/week	81	1 (ref.)		1 (ref.)	

All *p* values are given with logistic regression

*95% CI* 95% confidence interval

The OR for estimated 24 h UIE < 65 µg/day according to iodine-rich sources are shown in Table 4. Intake of dairy products (OR 3.59, 95% CI 1.13–11.43, *p* = 0.031) and sweet milk (OR 2.77, 95% CI 1.37–5.61, *p* = 0.005) < 2 times/day versus ≥ 2 times day was associated with estimated 24 h UIE < 65 µg/day. Similar findings were observed after adjustments (Table 4).

**Table 4:** Odds ratio (OR) for estimated 24 h UIE < 65 µg/day (median) according to intake of seafood, dairy products and eggs

Dietary products	*N*	Unadjusted		Adjusted for gender, parental education	
		OR (95% CI)	*p* value	OR (95% CI)	*p* value
Seafood dinner (all)					
< 2 times/week	103	0.89 (0.51–1.55)	0.670	1 (ref.)	0.942
≥ 2 times/week	95	1 (ref.)		0.98 (0.55–1.75)	
Seafood (spreads)					
< 2 times/week	132	0.69 (0.38–1.26)	0.229	0.66 (0.36–1.20)	0.173
≥ 2 time/week	66	1 (ref.)		1 (ref.)	
Dairy products (all)					
< 2 times/day	102	3.59 (1.13–11.43)	0.031	4.05 (1.23–13.28)	0.021
≥ 2 times/day	96	1 (ref.)		1 (ref.)	
Sweet milk					
< 2 times/day	45	2.77 (1.37–5.61)	0.005	2.82 (1.38–5.76)	0.004
≥ 2 times/day	153	1 (ref.)		1 (ref.)	
Cultured milk and yoghurt					
< 4 times/week	90	1.64 (0.93–2.87)	0.088	1.61 (0.90–2.88)	0.108
≥ 4 time/week	108	1 (ref.)		1 (ref.)	
White cheese					
< 4 times/week	94	0.87 (0.50–1.51)	0.617	0.95 (0.54–1.68)	0.864
≥ 4 time/week	105	1 (ref.)		1 (ref.)	
Brown cheese					
< 4 times/week	153	1.19 (0.61–2.31)	0.611	1.23 (0.62–2.42)	0.558
≥ 4 time/week	45	1 (ref.)		1 (ref.)	
Eggs					
< 2 times/week	117	0.88 (0.50–1.56)	0.665	0.91 (0.51–1.63)	0.750
≥ 2 times/week	81	1 (ref.)		1 (ref.)	

All *p* values are given with logistic regression

*95% CI* 95% confidence interval

## Discussion

In this study, we have examined iodine status of preschool children in Bergen, Norway. This population was found to be iodine sufficient with a median UIC of 132 µg/L. Low intake of sweet milk and dairy products were significant predictors of low iodine status.

According to WHO, median UIC between 100 and 199 µg/L in school-aged children defines a population with no iodine deficiency, in addition not more than 20% should have UIC below 50 µg/L [23]. Even though the median of the UIC is in the sufficiency range, 30% of the children had UIC below 100 µg/L and 17% had I/Cr ratio below 100 µg/g. Our UIC results are in accordance with other studies. In a recent Norwegian study including 47 children (aged 3–9 years), the median UIC was 148 µg/L [11]. In a study performed in 279 Australian preschool children (aged 1–5 years), the median UIC was 129 µg/L [24]. In addition, in a Portuguese study in school children (aged 6–12 years) the median UIC was 129 µg/L. However, in the latter study the median UIC in boys was significantly higher than in girls, which was partially explained by a higher energy intake among boys [25]. Similarly, in 857 school-age children (6–12 years old) from Sweden, the median UIC was 125 µg/L and no gender differences were found [26]. In the present study, the energy intake could unfortunately not be estimated. Further, our values are in agreement with a study from Denmark examining the iodine status of pregnant women, their partners and their children (*N* = 51, age 6 years), were the children had a median UIC of 126 µg/L [27]. These agreements are interesting considering that, in contrast to Norway, the largest dietary source of iodine in the Swedish diet is iodized salt [26], and in Denmark, salt and bread have been fortified with iodine for the last 15 years [28]. However, the analytical methods in the Danish study were different, and the results may therefore not be directly comparable. Iodization of salt is not mandatory in Norway. In the present study, UIC < 100 µg/L was associated with low intake of sweet milk and estimated 24 h UIE < 65 µg/day with low intake of both sweet milk and dairy products in general. The role of dairy products in relation to adequate UIC is also in agreement with studies conducted in Norwegian adults [4, 16]. Studies from other countries have also found milk and dairy products to be important predictors of iodine status in children [29]. We did not find any associations between UIC and intake of seafood as dinner or spread, nor with cheese, eggs or cultured milk and yoghurt. Even though we did not find any associations between UIC or estimated 24 h UIE and seafood it is well known that lean fish, in general, is a good source of iodine if consumed. Fatty fish and most seafood spreads do not have such a high content of iodine. Nevertheless, fish is consumed less frequent than milk and other dairy products, and thus we do not necessarily expect to find an association with iodine measured in spot samples as such samples will contain iodine excreted only from the most recent intake. Still, a weekly intake of lean fish in concordance with the Norwegian recommendations will be expected to be an important contribution for individual iodine status.

Due to traditionally higher iodine content in milk and dairy products during winter, the children’s iodine concentration may have been affected, and consequently resulted in a somewhat higher iodine status than summer spot urine samples might have. Further, low intakes and/or the large variations in iodine content in different foods may be a possible explanation for the lack of further associations. Iodine content between dairy products, and between and within different fish species varies [30, 31]. However, regarding seasonal variation in milk, more recent data indicate less variation throughout the year [31].

The UIC measured in spot urine samples is a reflection of recent dietary iodine intake and is still the recommended biomarker for assessing iodine status in a population [22]. However, there is an ongoing debate on whether this is the best estimate for measuring iodine status, especially on an individual level [13]. The I/Cr ratio aims to reduce the effect of hydration in urinary iodine analysis [13, 32]. It has been discussed whether the method of relating urinary iodine to creatinine is too burdensome, expensive and unnecessary [22]. Urinary I/Cr ratios may be unreliable especially when protein intake is low, and loss of muscle mass will lead to reduced creatinine excretion in urine [13, 22]. However, it has also been considered a more reliable measure of iodine status, due to the day-to-day variations of iodine intake and water consumption [13]. Norwegian reference intervals for urinary creatinine concentration measured in spot urine samples in children (4–6 years) are not established [33].

The relatively large sample size from the current study is recognized as a strength, and according to Andersen et al., a sample size of this magnitude makes it possible to estimate (with 95% CI) the iodine status from spot urine samples within a precision range of ± 7–10% [34]. However, data on the latest consumed meal and time of collection of urine samples were not registered, which possibly could affect the iodine content of the urine sample. Furthermore, both publicly and privately owned kindergartens were included in the present study, but adjustment for parental education (socioeconomic status) did not materially affect the results (Table 3), further, the response rate of 90% (from the FFQ), knowing that participation in the study was voluntary, is considered to be high [35, 36]. However, one cannot exclude a difference between included individuals and those who declined participation in the present study.

The FFQ method largely depends on the memory and perceptions of the respondents [37], and self-reported dietary data may be subject to memory lapses, misinterpretations and modifications to more socially desirable responses [38]. However, of healthy foods, over reporting is most common and therefore it should strengthen our findings between iodine and milk and dairy products.

## Conclusions

The present study shows that the iodine status of preschool children in Bergen, Norway was adequate. Low intake of sweet milk was associated with UIC < 100 µg/L and sweet milk and dairy products in general with estimated 24 h UIE < 65 µg/day.
